# The Association Between *CTLA-4, CD80/86*, and *CD28* Gene Polymorphisms and Rheumatoid Arthritis: An Original Study and Meta-Analysis

**DOI:** 10.3389/fmed.2021.598076

**Published:** 2021-02-02

**Authors:** Weixi Liu, Zhicheng Yang, Yan Chen, Haoyu Yang, Xiaoxian Wan, Xindie Zhou, Ruiping Liu, Yunkun Zhang

**Affiliations:** Department of Orthopaedics, The Affiliated Changzhou No.2 People's Hospital of Nanjing Medical University, Changzhou, China

**Keywords:** rheumatoid arthritis, polymorphisms, *CTLA-4*, *CD80/86*, *CD28*, meta-analysis

## Abstract

**Background:** Rheumatoid arthritis (RA) is related to several pivotal susceptibility genes, including *cytotoxic T-lymphocyte-associated protein 4 (CTLA-4)* and *costimulatory molecule (CD80/CD86)* genes. Although the connection between polymorphisms of *CTLA-4* and *CD86* genes in different populations of RA have been studied extensively, the results are controversial.

**Objective:** To clarify the correlation in the Chinese Han population between *CTLA-4, CD80/86*, and *CD28* gene polymorphisms, and RA susceptibility.

**Methods:** A case-control study (574 RA patients and 804 controls) was conducted to determine the correlation between *CTLA-4* rs231775 and rs16840252 gene polymorphisms, *CD86* rs17281995 gene polymorphisms, and the risk of RA for the Chinese Han population. Furthermore, an additional meta-analysis, including three single nucleotide polymorphisms (SNPs) (*CTLA-4* rs231775, *CTLA-4* rs3087243, and *CTLA-4* rs5742909) from 32 citations, including 43 studies, 24,703 cases and 23,825 controls was performed to elucidate the relationship between known SNPs in the *CTLA-4* genes and RA for more robust conclusions.

**Results:** The results showed that *CTLA-4* rs231775 gene polymorphism decreased the RA risk (GA vs. AA, OR = 0.77, *P* = 0.025), whereas *CTLA-4* rs16840252 and *CD86* rs17281995 gene polymorphisms were not related to RA susceptibility. Stratification analyses by RF, ACPA, CRP, ESR, DAS28, and functional class identified significant associations for *CTLA-4* rs231775 and rs16840252 gene polymorphisms in the RF-positive and RF-negative groups. A meta-analysis of the literature on *CTLA-4* gene polymorphisms and RA risk revealed that the risk of RA was decreased by *CTLA-4* rs231775 gene polymorphisms.

**Conclusions:** The *CTLA-4* rs231775 gene polymorphism decreased the risk of RA, whereas *CTLA-4* rs16840252 and *CD86* rs17281995 gene polymorphisms were not related to RA risk. A meta-analysis indicated that *CTLA-4* rs231775 and rs3087243 gene polymorphisms decreased the risk of RA. To support these analytical results, additional clinical cases should be investigated in further studies.

## Introduction

Rheumatoid arthritis (RA) is an autoimmune disease characterized by persistent inflammation of the synovial membranes of joints and progressive cartilage damage, ultimately leading to severe joint deformity and loss of function (1). According to current research on RA pathogenesis and etiology, 50–60% of its susceptibility is attributed to genetic factors (2). Recently, RA-related genes have been studied intensively, including *CTLA-4, CD80/CD86*, and *CD28* genes.

**Figure 1:**
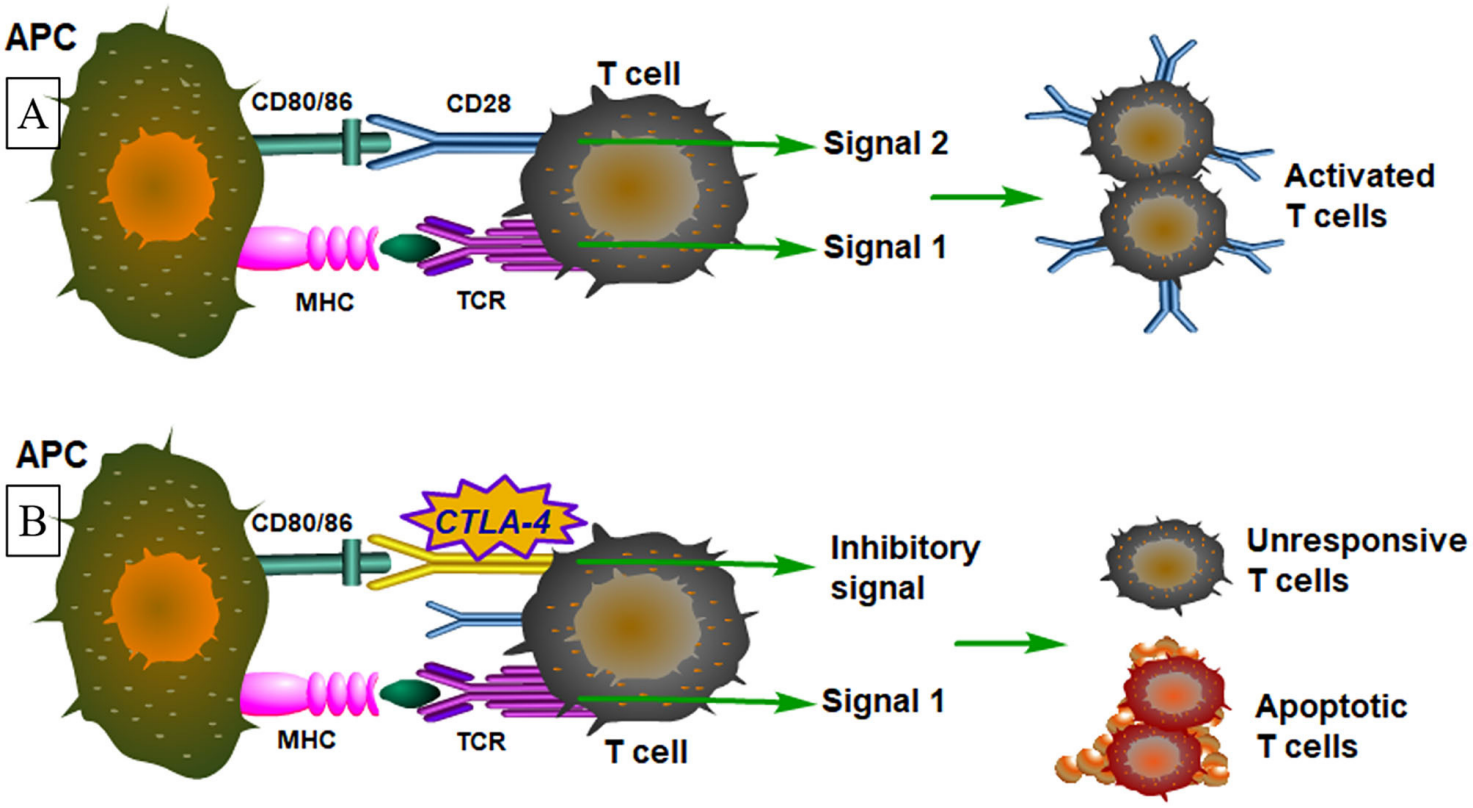
An illustrative figure shows the immunological mechanism of T cell activation. **(A)** Signal 1 means T cells are activated by peptide–MHC antigen and the T-cell antigen receptor (TCR) delivers activating signals. Antigen presenting cells (APCs) express costimulatory molecules that bind to receptors on the T-cell simultaneously with antigen recognition. In signal 2, the costimulatory receptors deliver signals, that cooperate with TCR signals to activate the T cell. **(B)** In a continuous immune response, the expression of CTLA-4 is upregulated which delivers signals that block the activating signals delivered by the T-cell antigen receptor (signal 1 plus coinhibitory signals). The main function will be either functional inactivation of the T cells or T-cell apoptosis.

T lymphocytes play an essential role in RA and their activation not only requires recognition of specific antigenic peptides by the T-cell receptor (TCR), but also the co-stimulatory signals provided by accessory surface molecules on T cells (3). CD28, a co-stimulatory receptor, is consecutively expressed on resting T-cells. CTLA-4, a co-inhibitory receptor, is a member of the CD28 family expressed on the surface of T cells soon after its activation (3, 4). CD80 and CD86 (B7 molecules), mainly expressed on B cells, monocytes/macrophages, and dendritic cells, are the ligands of CD28 and CTLA-4, which are upregulated when activated. The binding of the ligands and CD28 promotes the activation of a TCR-stimulated T cell, while the binding of CTLA-4 causes the inhibition of T cell activation (5). CTLA-4 has higher affinity with CD80-CD86 than CD28, although it is homologous to CD28 (6). In a continuous immune response, the expression of CTLA-4 is upregulated to inhibit the proliferation of T-cell and reduce interleukin (IL)-2 production. For B7 molecules, the main function is to augment and sustain T-cell responses by interacting with CD28, while they can also provide inhibitory signals when binding with CTLA-4 (7). The immunological mechanism of T cell activation is illustrated in Figure 1. Lack of CTLA-4 will therefore cause severe lymphoproliferation and harmful destruction of multiorgan tissues, indicating that it plays an vital role in negative regulatory functions (8). Hence, changes in *CTLA4* and *CD86* genes have the potential to increase the immune response of autoreactive T-cells to self-antigens (9). In mouse experiments, Ewing et al. (10) confirmed that T-cell co-stimulation by connecting with CD28 and its negative regulator CTLA-4 played an important role in accelerating atherosclerosis development. According to recent studies, molecular variants of CTLA-4 were discovered in a number of autoimmune and inflammatory diseases mediated by T-cells, especially in RA (9, 11, 12).

The association between *CTLA-4, CD80/86*, and *CD28* gene polymorphisms and RA susceptibility may offer new research possibilities to conduct further studies on RA. Although many studies (5, 13–44) have discovered the association between polymorphisms in the *CTLA-4* and *CD86* genes, the results are contradictory and inconclusive. As different populations have various lifestyles, gene pools and gene-environment interactions, it is impossible to expect that the risk of RA in each population is the same as genotypes. Here, a case-controlled study was conducted to reveal the association between the *CTLA-4* rs231775 gene polymorphism, *CTLA-4* rs16840252 gene polymorphism, *CD86* rs17281995 gene polymorphism, and RA risks in the Chinese Han population. Considering a single case-controlled study may lead to inconclusive results and be less persuasive because of clinical heterogeneity, limited sample sizes, and different ethnic populations, an additional meta-analysis was performed to verify the relevance between known SNPs in the *CTLA-4, CD80/86*, and *CD28* genes and RA to obtain more robust conclusions.

## Patients and Methods

### Study Population

This study was conducted in accordance with the Helsinki Declaration, and was approved by the Ethics Committee of the Affiliated Changzhou No. 2 People's Hospital of Nanjing Medical University.

In this study, 574 RA patients (427 women and 147 men), diagnosed using the 2010 ACR/EULAR classification standard for RA (45), were recruited successively from the Affiliated Changzhou No. 2 People's Hospital of Nanjing Medical University, the Changzhou First Hospital, and the Changzhou Traditional Chinese Medical Hospital (between September 2010 and October 2013). Exclusion criteria included patients from other countries, with other autoimmune diseases, a current or previous history of malignancy, other major systemic diseases, or a family history of autoimmune diseases. The 804 controls (599 women and 205 men) were recruited from the identical institutions during the same period and the majority of them were trauma patients matched for age and sex without RA and other autoimmune diseases. After providing written informed consent, every patient was interviewed separately by using a pre-tested questionnaire to obtain relevant data related to related risk and demographics elements.

### Genomic DNA Extraction and Genotyping

All blood samples were preserved by ethylenediaminetetraacetic acid tubes. QIAamp DNA blood mini kit (Qiagen, Hilden, Germany) was performed to extracted genomic DNA from whole blood. The MassARRAY system (Sequenom, San Diego, California, USA) and matrix-assisted laser desorption/ionization time-of-flight mass spectrometry were used to genotype SNPs, as previously described (46).

### Power Analysis

To evaluate the statistical power of this study, we used the Power and Sample Size Calculations, Version 3.1.6. for power analysis. Our study was performed with the power calculated with a significant value of 0.05 (47).

### Statistical Analysis

To assess the correlation between the studied SNPs and RA risk, we calculated the odds ratio (OR) and 95% confidence interval (CI) for five gene models (allele, dominant, recessive, homozygous, and heterozygous). These statistical analyses were all performed using SAS software package (ver. 9.1.3; SAS Institute, Cary, NC, USA) with a significance level of *P* < 0.05. By applying the goodness-of-fit χ^2^-test to contrast the observed and expected genotype frequencies among controls, we analyzed the Hardy-Weinberg equilibrium (HWE) of the genotypes.

### Meta-Analysis

To thoroughly characterize the association of SNPs in the *CTLA-4, CD80/86*, and *CD28* genes with RA, we searched the databases of Medline, PubMed, Embase, and the Cochrane Library to identify published studies through December 2018. The key words of “polymorphism,” “SNP,” “CTLA-4,” “CD80/86,” “CD28,” “rheumatoid arthritis,” and “RA” were combined for free research. No language or other restrictions were placed on the search. The reference lists of all the related papers were examined to identify any initially omitted studies.

The selection criteria were as follows:

- Studies that focused on humans;
- Studies that evaluated the association between SNPs of *CTLA-4, CD80/86*, and *CD28* genes and RA;
- Studies that included detailed genotype data.

Studies were excluded based on the following exclusion criteria:

- Duplication of previous publications;
- Review, editorial or other types of studies, which were not focused on detailed genotype research;
- Studies that failed to obtain detailed genotype data.

Studies were not conducted on patients who had cancer or other diseases that might have influenced the results. Any studies with questionable inclusion/exclusion criteria were discussed and disagreements were resolved by two reviewers independently evaluating the methodological quality of the included studies. The statistical analyses in our groups were performed using Stata 11.0 software (StataCorp, College Station, TX, USA). Their high methodological quality was evaluated by the Newcastle-Ottawa Scale (NOS) scores. For each meta-analysis, the pooled odds ratio (OR) was calculated for each gene variant, and 95% confidence intervals (CI) were established.

## Results

### Clinical Details of the Study Population

The clinical characteristics of all patients are summarized in Table 1. In this study, cases and controls were clearly matched for age and sex (*P* = 0.080 and *P* = 0.962, respectively). The observed frequency distributions of the rs231775, rs16840252, and rs17281995 genotypes in the control population and the RA patients, which conformed to the HWE. respectively, are presented in Table 2.

**Table 1:** Patient demographics and risk factors in rheumatoid arthritis.

**Variable**	**Cases (*n* = 574)**	**Controls (*n* = 804)**	***P***
Age (years)	54.5 (± 15.1)	55.7 (±10.1)	0.080
Female/male	427/147	599/205	0.962
Age at onset, years, mean ± SD	45.6 (±12.9)	—	—
Disease duration, years, mean SD	8.9 (±9.2)	—	—
Treatment duration, years, mean ± SD	7.6 (±7.8)	—	—
RF-positive, no. (%)	456 (79.4%)	—	—
ACPA positive, no. (%)	300 (52.2%)	—	—
CRP-positive, no. (%)	323 (56.3%)	—	—
ESR, mm/h	33.7 (±25.2)	—	—
DAS28	4.3 (±1.5)	—	—
**Functional class, no. (%)**			
I	73 (12.7%)	—	—
II	256 (44.6%)	—	—
III	209 (36.4%)	—	—
IV	36 (6.3%)	—	—

*RF, rheumatoid factor; ACPA, anti-cyclic citrullinated peptide antibodies; CRP, C-reactive protein; ESR, erythrocyte sedimentation rate; DAS28, RA disease activity score*.

**Table 2:** Logistic regression analysis of association between *CTLA-4* rs231775, *CTLA-4* rs16840252 and *CD86* rs17281995 polymorphisms and the risk of rheumatoid arthritis.

**Genotype**	**Cases (*****n*** **=** **574)**		**Controls (*****n*** **=** **804)**		**OR (95% CI)**	***P***
	***n***	**%**	***n***	**%**		
**CTLA-4 rs231775**						
G vs. A	332/812	29.0/71.0	478/1,116	30.0/70.0	0.96 (0.81, 1.13)	0.585
GG+GA vs. AA	269/303	47.0/53.0	410/387	51.4/48.6	0.84 (0.68, 1.04)	0.107
GG vs. GA+AA	63/509	11.0/89.0	68/729	8.5/91.5	1.33 (0.93, 1.90)	0.125
GG vs. AA	63/303	11.0/53.0	68/387	8.5/48.6	1.18 (0.81, 1.72),	0.378
GA vs. AA	206/303	36.0/53.0	342/387	42.9/48.6	**0.77 (0.61, 0.97)**	**0.025**
**CTLA-4 rs16840252**						
T vs. C	158/988	13.8/86.2	223/1,361	14.1/85.9	0.98 (0.78, 1.22)	0.828
TT+TC vs. CC	145/428	25.3/74.7	205/587	25.9/74.1	0.97 (0.76, 1.24)	0.809
TT vs. TC+CC	13/560	2.3/97.7	18/774	2.3/97.7	0.99 (0.49, 2.05)	0.996
TT vs. CC	13/428	2.3/74.7	18/587	2.3/74.1	0.99 (0.48, 2.04)	0.979
TC vs. CC	132/428	23.0/74.7	187/587	23.6/74.1	0.96 (0.75, 1.25)	0.804
**CD86 rs17281995**						
C vs. G	61/1,083	5.3/94.7	98/1,508	6.1/93.9	0.87 (0.62, 1.20)	0.394
CC+GC vs. GG	60/512	10.5/89.5	96/707	12.0/88.0	0.86 (0.61, 1.22)	0.399
CC vs. GC+GG	1/571	0.2/99.8	2/801	0.2/99.8	0.70 (0.06, 7.75)	0.772
CC vs. GG	1/512	0.2/89.5	2/707	0.2/88.0	0.69 (0.06, 7.64)	0.763
GC vs. GG	59/512	10.3/89.5	94/707	11.8/88.0	0.87 (0.61, 1.22)	0.416

*Bold values are statistically significant (P < 0.05)*.

### Association Between *CTLA-4* Gene Polymorphisms and RA Risk

The genotypic distributions of the *CTLA-4* rs231775 gene polymorphisms in the subjects are presented in Table 2. The results showed that the GA genotype was associated with a decreased risk of RA (GA vs. AA, OR = 0.77, *P* = 0.026) using logistic regression analyses. The effects on RA risk of this SNP were further evaluated according to the characteristics of rheumatoid factor (RF), anti-cyclic citrullinated peptide antibodies (ACPA), C-reactive protein (CRP), erythrocyte sedimentation rate (ESR), RA disease activity score (DAS28), and function class. The American College of Rheumatology's improved grading standards were the following: level 1: life, work, and competitive sports are not restricted; level 2: life, no restrictions on work, and no restrictions on competitive sports; level 3: life is unrestricted, work and competitive sports are restricted; level 4: life, work, and competitive sports are restricted. A significant association was detected among the RF-positive and RF-negative groups (GG, OR = 2.53, *P* = 0.002; AG+GG, OR = 1.98, *P* = 0.019, Supplementary Table 1).

The genotypic distributions of the *CTLA-4* rs16840252 gene polymorphism in this population are well-delineated in Table 2. Logistic regression analyses suggested that the TT genotype, or T allele carriers of the rs16840252 polymorphism, were not related with the risk of RA (Table 2). The effects on RA risk of this SNP were further tested according to the characteristics of RF, ACPA, CRP, ESR, DAS28, and function class; significant associations were detected among the RF-positive and RF-negative groups, which suggested that the TT genotype of rs16840252 polymorphisms might be associated with the expression of RF (Supplementary Table 1).

### Association Between *CD86* rs17281995 Gene Polymorphism and RA Risk

The frequencies of the genotypes for the *CD86* rs17281995 gene in cases and controls are shown in Table 2. In this study, discrepancies of genotypes for the rs17281995 polymorphisms in the control subjects conformed to the HWE. Then, logistic regression analyses showed that the *CD86* rs17281995 gene polymorphisms had no relationship with RA risk. The effects on RA risk of this SNP were further assessed according to the characteristics of RF, ACPA, CRP, ESR, DAS28, and function class; no significant association was found (Supplementary Table 1).

### Power Analysis

The power of our study for *CTLA-4* rs231775, *CTLA-4* rs16840252, and *CD86* rs17281995 were 0.668, 0.057, and 0.088, respectively.

### Meta-Analysis: General Characteristics of the Included Studies and Quantitative Analysis

**Figure 2:**
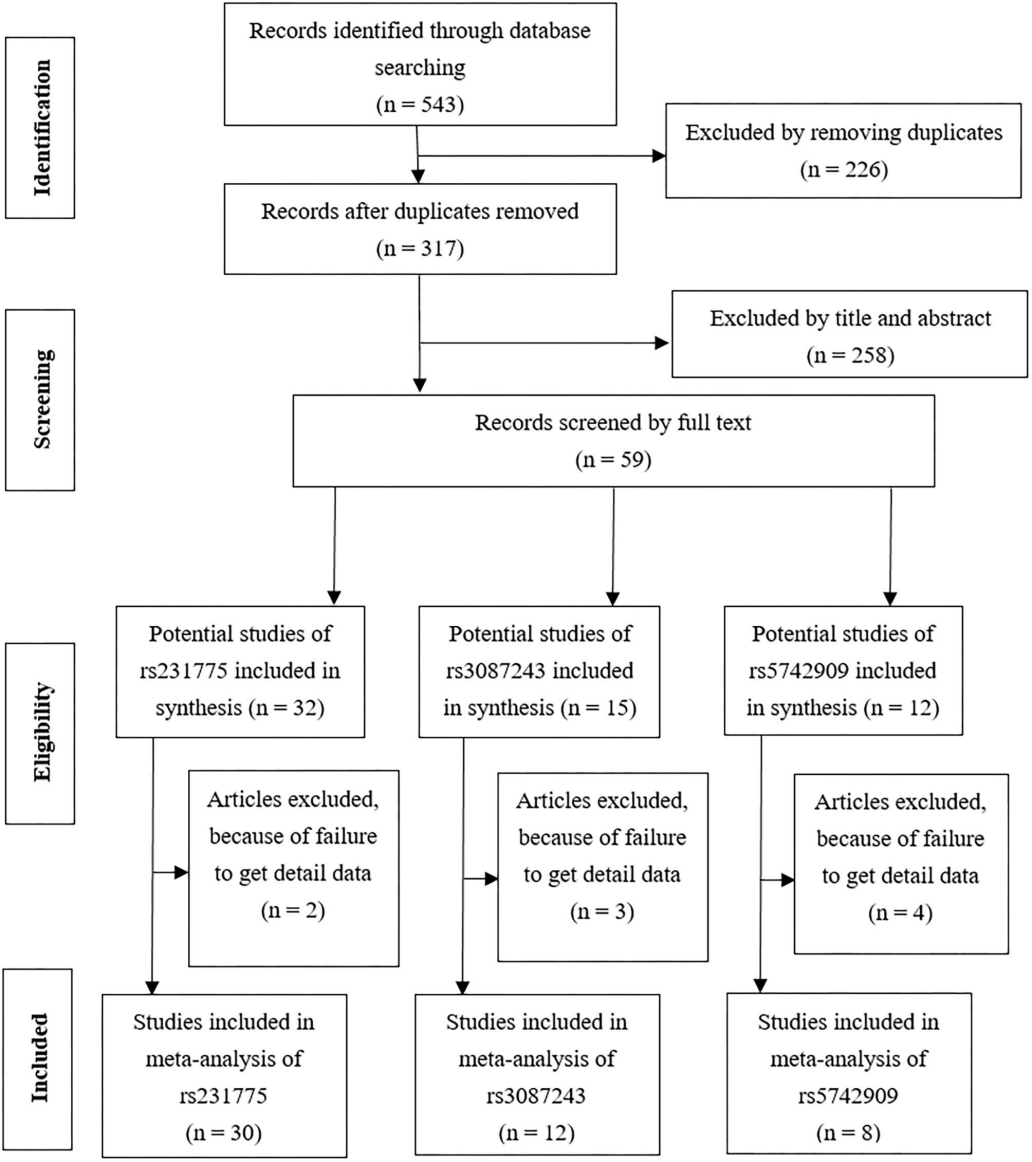
A flowchart shows the results of the literature search and selection for this study of CTLA-4.

A search of the literature yielded 543 citations. According to the application and refinement of the literature search strategy, 50 related studies were eligible for the data extraction. The flowchart of the reviews, which showed the selection protocol that we used for qualified studies, is presented in Figure 2. In this meta-analysis, these clinical characteristics of the studies exploring the association between the SNPs of *CTLA-4, CD80/86*, and *CD28* genes and RA susceptibility are listed in Supplementary Table 2. A total of 38 citations including 21 SNPs and 75 studies were selected. Finally, three SNPs (*CTLA-4* rs231775, *CTLA-4* rs3087243, and *CTLA-4* rs5742909, Supplementary Table 3) were incorporated into our meta-analysis [32 citations including 43 studies, 24,703 cases and 23,825 controls, (5, 13–43)] because a sufficient number of studies was identified (≥ 5). According to the NOS scores, the included articles ranged from 5 to 7 stars, showing their high methodological quality.

General analysis showed that the rs231775 polymorphism of the *CTLA-4* gene decreased RA risk [OR and 95% CI: 0.92 (0.86–0.98) in G vs. A; 0.86 (0.75–0.99) in GG+GA vs. AA; 0.86 (0.78–0.95) in GG vs. GA+AA; and 0.90 (0.83–0.98) in GG vs. AA, Table 3]. Stratification analysis showed that the *CTLA-4* gene rs231775 polymorphism decreased the risk of RA among Asians, Caucasians, and Latin-Americans but not among Africans (Table 3).

**Table 3:** Meta-analysis of the association between *CTAL-4* gene polymorphisms and rheumatoid arthritis risk.

**Comparison**	**Category**	**Category**	**Studies**	**OR (95% CI)**	***P*-value**	***P* for heterogeneity**
**CTLA-4 rs231775**						
G vs. A	Total		30	**0.92 (0.86, 0.98)**	**0.015**	<0.001
	Ethnicity	Asian	12	**0.88 (0.83, 0.94)**	** <0.01**	0.446
		Caucasian	16	0.93 (0.84, 1.04)	0.201	<0.001
		Latin-American	1	**0.69 (0.52, 0.91)**	**0.010**	–
		African	1	1.25 (0.81, 1.94)	0.318	–
GG+GA vs. AA	Total		30	0.95(0.85, 1.07)	0.406	<0.001
	Ethnicity	Asian	12	**0.86 (0.75, 0.99)**	**0.041**	0.043
		Caucasian	16	1.02(0.85, 1.23)	0.821	0.001
		Latin-American	1	0.77 (0.47, 1.27)	0.308	–
		African	1	1.33 (0.72, 2.46)	0.356	–
GG vs. GA+AA	Total		30	**0.86 (0.78, 0.95)**	**0.004**	0.001
	Ethnicity	Asian	12	0.87 (0.75, 1.01)	0.065	0.270
		Caucasian	16	**0.87 (0.76, 0.99)**	**0.039**	0.001
		Latin-American	1	**0.53 (0.35, 0.81)**	**0.003**	–
		African	1	1.30 (0.57, 2.97)	0.534	–
GG vs. AA	Total		30	**0.90 (0.83, 0.98)**	**0.014**	0.001
	Ethnicity	Asian	12	**0.80 (0.69, 0.91)**	**0.001**	0.479
		Caucasian	16	0.99 (0.88, 1.10)	0.797	0.001
		Latin-American	1	**0.54 (0.30, 0.94)**	**0.031**	–
		African	1	1.48 (0.60, 3.62)	0.392	–
GA vs. AA	Total		30	1.00 (0.88, 1.14)	0.992	<0.001
	Ethnicity	Asian	12	0.89 (0.75, 1.05)	0.162	0.005
		Caucasian	16	1.10 (0.90, 1.33)	0.350	0.001
		Latin-American	1	1.01 (0.59, 1.72)	0.980	–
		African	1	1.29 (0.67, 2.48)	0.449	–
**CTLA-4 rs3087243**						
A vs. G	Total		12	**0.88 (0.85, 0.92)**	** <0.001**	0.092
	Ethnicity	Caucasian	9	**0.89 (0.85, 0.93)**	** <0.001**	0.089
		Latin-American	1	0.79 (0.60, 1.06)	0.112	–
		Asian	2	**0.82 (0.72, 0.95)**	** <0.007**	0.134
AA+AG vs. GG	Total		12	**0.83 (0.78, 0.88)**	** <0.001**	0.040
	Ethnicity	Caucasian	9	**0.84 (0.79, 0.89)**	** <0.001**	0.083
		Latin-American	1	**0.63 (0.42, 0.96)**	**0.029**	–
		Asian	2	**0.79 (0.66, 0.95)**	**0.011**	0.035
AA vs. AG+GG	Total		12	**0.88 (0.82, 0.95)**	**0.001**	0.768
	Ethnicity	Caucasian	9	**0.89 (0.82, 0.96)**	**0.002**	0.591
		Latin-American	1	0.96 (0.56, 1.65)	0.891	–
		Asian	2	0.76 (0.55, 1.06)	0.104	0.985
AA vs. GG	Total		12	**0.79 (0.73, 0.86)**	** <0.001**	0.329
	Ethnicity	Caucasian	9	**0.80 (0.74, 0.88)**	** <0.001**	0.208
		Latin-American	1	0.72 (0.40, 1.31)	0.285	–
		Asian	2	**0.67 (0.48, 0.94)**	**0.020**	0.479
AG vs. GG	Total		12	**0.84 (0.79, 0.90)**	** <0.001**	0.076
	Ethnicity	Caucasian	9	**0.85 (0.80, 0.91)**	** <0.001**	0.193
		Latin-American	1	**0.61 (0.39, 0.94)**	**0.024**	–
		Asian	2	**0.81 (0.67, 0.98)**	**0.029**	0.031
**CTLA-4 rs5742909**						
T vs. C	Total		8	1.19 (0.88, 1.62)	0.255	<0.001
	Ethnicity	Caucasian	4	1.22 (0.80, 1.86)	0.365	0.004
		Latin-American	1	1.00 (0.53, 1.89)	1.000	–
		Asian	3	1.14 (0.54, 2.42)	0.733	0.003
TT+TC vs. CC	Total		8	1.25 (0.86, 1.80)	0.245	<0.001
	Ethnicity	Caucasian	4	1.28 (0.79, 2.07)	0.323	0.003
		Latin-American	1	0.89 (0.46, 1.74)	0.733	–
		Asian	3	1.26 (0.50, 3.19)	0.625	0.001
TT vs. TC+CC	Total		8	1.43 (0.90, 2.28)	0.128	0.519
	Ethnicity	Caucasian	4	1.46 (0.77, 2.78)	0.245	0.394
		Latin-American	1	5.05 (0.24, 105.86)	0.297	–
		Asian	3	1.27 (0.63, 2.55)	0.502	0.224
TT vs. CC	Total		8	1.71 (0.98, 2.73)	0.123	0.302
	Ethnicity	Caucasian	4	1.62 (0.86, 3.07)	0.136	0.218
		Latin-American	1	4.95 (0.24, 103.73)	0.303	–
		Asian	3	1.69 (0.84, 3.43)	0.143	0.131
TC vs. CC	Total		8	1.25 (0.86, 1.81)	0.239	<0.001
	Ethnicity	Caucasian	4	1.27 (0.81, 1.99)	0.289	0.010
		Latin-American	1	0.79 (0.40, 1.58)	0.505	–
		Asian	3	1.33 (0.52, 3.41)	0.553	0.001

*Bold values are statistically significant (P < 0.05)*.

The meta-analysis also indicated that the *CTLA-4* gene rs3087243 polymorphism decreased RA risk [OR and 95% CI: 0.88 (0.85–0.92) in A vs. G; 0.83 (0.78–0.88) in AA+AG vs. GG; 0.88 (0.82–0.95) in AA vs. AG+GG; 0.79 (0.73–0.86) in AA vs. GG; and 0.84 (0.79–0.90) in AG vs. GG, Table 3]. Stratification analysis showed that the *CTLA-4* gene rs3087243 polymorphism decreased the risk of RA among Asians, Caucasians, and Latin-Americans (Table 3). No significant association was found between the *CTLA-4* gene rs5742909 polymorphism and RA risk.

The results did not change after eliminating studies (32, 35, 42), which did not meet HWE, suggesting that all data arising from the meta-analysis were trustworthy and stable. These results of sensitivity analysis also indicated that the data were stable and credible. Finally, Egger's and Begg's tests did not reveal publication bias.

## Discussion

In our study, we conducted a meta-analysis to determine whether SNPs in the *CTLA-4, CD80/86*, and *CD28* genes were associated with RA susceptibility. In addition, our meta-analysis was conducted by combining eight published case-control studies with our study to provide more reliable conclusions. The results indicated that both *CTLA-4* rs231775 and rs3087243 reduced the risk of RA. Previously, five meta-analyses (18, 48–51) reported the relationship between *CTLA-4* gene polymorphisms and the risk of RA. (1) Daha et al. (51) revealed that *CTLA4* gene rs3087243 polymorphisms decreased the risk of RA among East Asians and Caucasians. After including studies from 2009, the present meta-analysis confirmed that rs3087243 polymorphisms decreased the RA susceptibility in Asians, Caucasians, and Latin-Americans populations. (2) Tang et al. (18), Li et al. (49), and Lee et al. (50) all reported that *CTLA4* gene rs231775 polymorphisms increased the risk of RA, which was contrary to our results. All studies included in the above three meta-analyses were updated to 2013, whereas the present meta-analysis included five additional case-controlled studies (increased to 1,433 cases and 1,842 controls). Unbiased epidemiological studies and massive samples of the predisposition of gene polymorphisms would offer a more profound understanding of the correlation between diseases and candidate genes. (3) Lee et al. (48) suggested that the *CTLA-4* gene rs5742909 was not associated with RA risk, which was not consistent with our findings. In addition to including more citations, we could not extract valid data from one study (32) by Lee et al. Sensitivity analysis indicated that the present data on the three SNPs was trustworthy and robust.

To further characterize the role of CD28-CD80/CD86 and CTLA-4 in the development of RA, our study was initiated. The results indicated that *CTLA-4* rs231775 gene polymorphisms decreased the risk of RA, whereas *CTLA-4* rs16840252 and *CD86* rs17281995 gene polymorphisms were not related to RA risk. In the pathogenesis of RA, genetic and environmental factors are apparently implicated. CTLA-4 impedes IL-2 production and IL-2 receptor expression, and inhibits the function of cytotoxic T-lymphocytes as a vital co-stimulatory molecule. There are two main mRNA transcripts in humans produced by the *CTLA-4* gene. CTLA-4 is known as a homolog to CD28. When an immune response continues, CTLA-4 is upregulated and competes with CD28, leading to reduction of IL-2 production and inhibition of T-cell proliferation (52). RA is a complicated disease, and its clinical heterogeneity may account for many additional factors, including the decrease or existence of RF. Our stratification research indicated that the rs231775 TT genotypes and the rs16840252 GG genotypes were more significantly correlated with RF-positive subgroups than with RF-negative subgroups. The data suggested that these genotypes of *CTLA-4* gene polymorphisms associated with RA risk might not be associated with a higher risk of yielding rheumatoid factor (RF). Previous genetic association studies yielded conflicting results. Different geographical environments, sample sizes, and study designs may explain these discrepancies.

Previous studies have shown that a soluble, recombinant, and completely humanized fusion protein-Abatacept (CTLA-4Ig) can bind to CD80 and CD86 on antigen presenting cells (APC), thus, blocking interaction with CD28 on T cells (53). In clinical trials, an intravenous (IV) or subcutaneous (SC) Abatacept regimen has beneficial effects on RA signs and symptoms, disease activity, structural damage progression, and body function. Moreover, the current evidence suggested that Abatacept was a useful treatment option for patients with rheumatoid arthritis (54). Therefore, CTLA-4 could be used as a therapeutic target for RA, which proved that the coding gene of CTLA-4 is related to the risk of RA.

There were some limitations in our study. First, due to the lack of corresponding data, subgroups of some confounding factors could not be analyzed. Second, owing to confounding factors, the results of our research were based on unadjusted evaluations. Third, our research cohorts were merely Asians, so it is necessary to study other racial groups. Fourth, due to limited sample sizes, the conclusions obtained by stratification analyses of the rs231775/rs16840252/rs17281995 polymorphisms should be interpreted cautiously. Fifth, it is essential for researchers to investigate clinical cases in further studies to support our results. Finally, all these five genetic models of inheritance were used in the study; hence, type I errors may have appeared as lacking correction for multiple testing.

In conclusion, based on our retrospective analyses, the *CTLA-4* rs231775 gene polymorphisms decreased the risk of RA, whereas the *CTLA-4* rs16840252 and *CD86* rs17281995 gene polymorphisms were not related to RA risk. The present meta-analysis indicated that *CTLA-4* rs231775 and rs3087243 gene polymorphisms decreased the risk of RA. Additional investigation of clinical cases should be performed to verify these analytical results.

## Data Availability Statement

The original contributions presented in the study are included in the article/Supplementary Materials, further inquiries can be directed to the corresponding authors.

## Ethics Statement

The studies involving human participants were reviewed and approved by Ethics Committee of the Affiliated Changzhou No.2 People's Hospital of Nanjing Medical University. The patients/participants provided their written informed consent to participate in this study. Written informed consent was obtained from the individual(s) for the publication of any potentially identifiable images or data included in this article.

## Author Contributions

RL and YZ designed the study. ZY and YC collected samples. HY and XW conceived and conducted the experiments. RL, WL, and XZ analyzed the data and wrote the manuscript. All authors reviewed the manuscript and gave final approval for the work.

## Conflict of Interest

The authors declare that the research was conducted in the absence of any commercial or financial relationships that could be construed as a potential conflict of interest.

## Abbreviations

CTLA-4: cytotoxic T-lymphocyte-associated protein 4
CD: costimulatory molecule
RA: rheumatoid arthritis
OR: odds ratio
CI: confidence interval
HWE: Hardy-Weinberg equilibrium
SNP: single nucleotide polymorphism.
