# Supplementary material for: The Association Between CTLA-4, CD80/86, and CD28 Gene Polymorphisms and Rheumatoid Arthritis: An Original Study and Meta-Analysis
Source: Front Med (Lausanne). 2021 Feb 2;8:598076. doi: 10.3389/fmed.2021.598076 (PMC7884472; doi:10.3389/fmed.2021.598076)
Supplement: Supplementary file 1 [file Table_1.DOCX]

**Supplementary table 1** The association between rs16840252, rs17281995, rs231775 polymorphisms and clinical characteristics of RA

| Characteristics | Genotype distributions |  |  |  |
| --- | --- | --- | --- | --- |
| rs16840252 | CC | TC | TT | TC+TT |
| RF |  |  |  |  |
| Positive/negative | 346/82 | 103/29 | 7/6 | 110/35 |
| OR (95%CI); *P*-value | 1.0 (reference) | 0.84(0.52-1.36); 0.479 | **0.28(0.09-0.85); 0.016** | 0.75(0.48-1.17); 0.199 |
| ACPA |  |  |  |  |
| Positive/negative | 229/199 | 66/66 | 5/8 | 71/74 |
| OR (95%CI); *P*-value | 1.0 (reference) | 0.87(0.60-1.28); 0.481 | 0.54(0.18-1.69); 0.284 | 0.83(0.57-1.22); 0.344 |
| CRP |  |  |  |  |
| >10 mg/L /≤10 mg/L | 232/196 | 84/48 | 6/7 | 90/55 |
| OR (95%CI); *P*-value | 1.0 (reference) | 1.48(0.99-2.21); 0.056 | 0.72(0.24-2.19); 0.566 | 1.38(0.94-2.03); 0.099 |
| ESR |  |  |  |  |
| >25 mm/h/≤25 mm/h | 243/185 | 82/50 | 7/6 | 89/56 |
| OR (95%CI); *P*-value | 1.0 (reference) | 1.25(0.84-1.86); 0.277 | 0.89(0.29-2.69); 0.834 | 1.21(0.82-1.78); 0.332 |
| DAS28 |  |  |  |  |
| >3.2/≤3.2 | 320/108 | 96/36 | 7/6 | 103/42 |
| OR (95%CI); *P*-value | 1.0 (reference) | 0.90(0.58-1.40); 0.639 | 0.39(0.13-1.20); 0.090 | 0.83(0.54-1.26); 0.377 |
| Function class |  |  |  |  |
| Ⅲ+Ⅳ/Ⅰ+Ⅱ | 183/245 | 57/75 | 5/8 | 62/83 |
| OR (95%CI); *P*-value | 1.0 (reference) | 1.02(0.69-1.51); 0.931 | 0.84(0.27-2.60); 0.758 | 1.00(0.68-1.46); 1.000 |
| Characteristics | Genotype distributions |  |  |  |
| rs17281995 | GG | GC | CC | GC+CC |
| RF |  |  |  |  |
| Positive/negative | 406/106 | 48/11 | 1/0 | 49/11 |
| OR (95%CI); *P*-value | 1.0 (reference) | 1.14(0.57-2.27); 0.711 | NA | 1.16(0.59-2.31); 0.667 |
| ACPA |  |  |  |  |
| Positive/negative | 272/240 | 28/31 | 0/1 | 28/32 |
| OR (95%CI); *P*-value | 1.0 (reference) | 0.80(0.47-1.37); 0.409 | NA | 0.77(0.45-1.32); 0.343 |
| CRP |  |  |  |  |
| >10 mg/L /≤10 mg/L | 290/222 | 31/28 | 1/0 | 32/28 |
| OR (95%CI); *P*-value | 1.0 (reference) | 0.85(0.49-1.45); 0.548 | NA | 0.88(0.51-1.50); 0.625 |
| ESR |  |  |  |  |
| >25 mm/h/≤25 mm/h | 297/215 | 34/25 | 1/0 | 35/25 |
| OR (95%CI); *P*-value | 1.0 (reference) | 0.99(0.57-1.70); 0.955 | NA | 1.01(0.59-1.74); 0.961 |
| DAS28 |  |  |  |  |
| >3.2/≤3.2 | 378/134 | 45/14 | 0/1 | 45/15 |
| OR (95%CI); *P*-value | 1.0 (reference) | 1.14(0.61-2.14); 0.685 | NA | 1.06(0.57-1.97); 0.845 |
| Function class |  |  |  |  |
| Ⅲ+Ⅳ/Ⅰ+Ⅱ | 214/298 | 31/28 | 0/1 | 31/29 |
| OR (95%CI); *P*-value | 1.0 (reference) | 1.54(0.90-2.65); 0.114 | NA | 1.49(0.87-2.54); 0.144 |
| Characteristics | Genotype distributions |  |  |  |
| rs231775 | AA | AG | GG | AG+GG |
| RF |  |  |  |  |
| Positive/negative | 43/20 | 163/43 | 249/54 | 412/97 |
| OR (95%CI); *P*-value | 1.0 (reference) | 1.76(0.94-3.30); 0.075 | **2.53(1.38-4.64); 0.002** | **1.98(1.11-3.51); 0.019** |
| ACPA |  |  |  |  |
| Positive/negative | 35/28 | 106/100 | 159/144 | 265/244 |
| OR (95%CI); *P*-value | 1.0 (reference) | 0.85(0.48-1.50); 0.569 | 0.88(0.51-1.52); 0.656 | 0.87(0.51-1.47); 0.601 |
| CRP |  |  |  |  |
| >10 mg/L /≤10 mg/L | 35/28 | 122/84 | 165/138 | 287/222 |
| OR (95%CI); *P*-value | 1.0 (reference) | 1.16(0.66-2.05); 0.605 | 0.96(0.55-1.65); 0.873 | 0.96(0.5-1.65); 0.873 |
| ESR |  |  |  |  |
| >25 mm/h/≤25 mm/h | 39/24 | 118/88 | 175/128 | 293/216 |
| OR (95%CI); *P*-value | 1.0 (reference) | 0.83(0.46-1.47); 0.515 | 0.84(0.48-1.47); 0.543 | 0.84(0.49-1.43); 0.510 |
| DAS28 |  |  |  |  |
| >3.2/≤3.2 | 230/73 | 149/57 | 43/20 | 192/77 |
| OR (95%CI); *P*-value | 1.0 (reference) | 0.83(0.55-1.24); 0.364 | 0.68(0.38-1.23); 0.204 | 0.71(0.49-1.03); 0.073 |
| Function class |  |  |  |  |
| Ⅲ+Ⅳ/Ⅰ+Ⅱ | 27/36 | 92/114 | 126/177 | 218/291 |
| OR (95%CI); *P*-value | 1.0 (reference) | 1.08(0.61-1.90); 0.801 | 0.95(0.55-1.64); 0.852 | 0.99(0.59-1.70); 0.997 |

RA: rheumatoid arthritis; RF: rheumatoid factor; ACPA: anti-cyclic citrullinated peptide antibodies; CRP: C-reactive protein; ESR: erythrocyte sedimentation rate; DAS28: RA disease activity score. Bold values are statistically significant (*P* <0.05).
