# Supplementary material for: The Association Between CTLA-4, CD80/86, and CD28 Gene Polymorphisms and Rheumatoid Arthritis: An Original Study and Meta-Analysis
Source: Front Med (Lausanne). 2021 Feb 2;8:598076. doi: 10.3389/fmed.2021.598076 (PMC7884472; doi:10.3389/fmed.2021.598076)
Supplement: Supplementary file 2 [file Table_2.DOCX]

**Supplementary table 2** Characteristics of included studies

| SNP | Author | Year | Country | Case/Control | NOS | HWE | Association between this SNP and RA risk |
| --- | --- | --- | --- | --- | --- | --- | --- |
| *CTLA-4* rs231775 | This study | 2018 | China | 572/797 | 3-1-2 | Y 0.54 | Decreased the risk of RA |
|  | Luterek | 2017 | Poland | 422/338 | 3-1-2 | Y 0.58 | Increased the risk of RA |
|  | Elshazli | 2015 | Egypt | 112/122 | 3-1-2 | Y 0.74 | Increased the risk of RA |
|  | Liu | 2013 | China | 213/303 | 3-1-2 | Y 0.06 | Not related |
|  | Alfadhli | 2013 | Kuwait | 114/282 | 3-1-2 | Y 0.36 | Not related |
|  | Tang | 2013 | China | 1489/1200 | 3-1-2 | Y 0.05 | Increased the risk of RA |
|  | Benhatchi | 2011. | Slovakia | 91/51 | 3-1-2 | Y 0.53 | Not related |
|  | Plant (U) | 2010 | UK | 1004/2659 | 3-1-3 | Y 0.67 | Not related |
|  | Plant (Gr) | 2010 | Greece | 272/287 | 3-1-3 | Y 0.05 | Not related |
|  | Plant (Ge) | 2010 | Germany | 220/260 | 3-1-3 | Y 6.2 | Not related |
|  | Plant (F) | 2010 | France | 684/162 | 3-1-3 | Y 0.47 | Not related |
|  | Munoz | 2010 | Mexico | 199/199 | 3-1-2 | Y 0.08 | Increased the risk of RA |
|  | Walker | 2009 | Canada | 1140/1248 | 3-1-2 | Y 0.61 | Not related |
|  | Tsukahara | 2008 | Japan | 1490/448 | 3-1-2 | Y 0.09 | Increased the risk of RA |
|  | Suppiah | 2006 | UK | 289/475 | 3-1-2 | Y 0.57 | Increased the risk of RA |
|  | Takeuchi | 2006 | Japan | 100/104 | 3-1-2 | Y 0.63 | Not related |
|  | Lei | 2005 | China | 326/250 | 2-1-2 | Y 0.56 | Increased the risk of RA |
|  | Barton | 2004 | UK | 132/156 | 3-1-2 | Y 0.24 | Not related |
|  | Liu | 2004 | China | 65/81 | 3-1-2 | N 0.02 | Not related |
|  | Lee | 2003 | China | 186/203 | 3-1-2 | Y 0.13 | Increased the risk of RA |
|  | Vaidya | 2002 | UK | 123/349 | 3-1-2 | Y 0.83 | Increased the risk of RA |
|  | Lee | 2002 | Korea | 86/86 | 3-1-2 | Y 0.24 | Increased the risk of RA |
|  | Milicic | 2001 | UK | 421/452 | 3-1-2 | Y 0.74 | Not related |
|  | Hadj | 2001 | Tunisia | 60/150 | 3-1-2 | Y 0.33 | Not related |
|  | Barton(1) | 2000 | UK | 192/96 | 3-1-2 | Y 0.5 | Not related |
|  | Barton(2) | 2000 | Spain | 136/144 | 3-1-2 | Y 0.29 | Not related |
|  | Yanagawa | 2000 | Japan | 85/200 | 2-1-2 | Y 0.21 | Increased the risk of RA |
|  | Gonzalez | 1999 | Spain | 138/305 | 3-0-2 | N 0.02 | Increased the risk of RA among females |
|  | Matsushita | 1999 | Japan | 461/150 | 2-1-2 | Y 0.88 | Not related |
|  | Seidl | 1999 | Germany | 258/457 | 2-1-2 | Y 0.62 | Not related |
| *CTLA-4* rs3087243 | Luterek | 2017 | Poland | 422/338 | 3-1-2 | Y 0.13 | Not related |
|  | Torres | 2013 | Mexico | 200/200 | 3-1-2 | Y 0.23 | Decreased the risk of RA |
|  | Gabalawy | 2011 | Canada | 332/490 | 3-1-2 | Y 0.9 | Not related |
|  | Barton | 2009 | UK | 3669/3049 | 3-1-2 | Y 0.73 | Decreased the risk of RA |
|  | Walker | 2009 | Canada | 1140/1248 | 3-1-2 | Y 0.66 | Increased the risk of RA |
|  | Costenbader | 2008 | USA | 423/420 | 3-1-2 | Y 0.24 | Not related |
|  | Tsukahara | 2008 | Japan | 1498/441 | 3-1-2 | Y 0.42 | Not related |
|  | Lei | 2005 | China | 326/250 | 2-1-2 | Y 0.11 | Increased the risk of RA |
|  | Plenge(1) | 2005 | Sweden | 1505/878 | 3-1-2 | Y 0.12 | Not related |
|  | Plenge(2) | 2005 | USA | 828/845 | 3-1-2 | Y 0.57 | Increased the risk of RA |
|  | Orozco | 2004 | Span | 433/398 | 3-1-2 | Y 0.99 | Not related |
|  | Barton | 2004 | UK | 719/755 | 3-1-2 | Y 0.89 | Not related |
| *CTLA-4* rs5742909 | Fattah | 2017 | Egypt | 100/100 | 3-1-2 | Y 0.4 | Increased the risk of RA |
|  | Torres | 2013 | Mexico | 200/200 | 3-1-2 | Y 0.46 | Increased the risk of RA |
|  | Liu | 2013 | China | 213/304 | 3-1-2 | Y 0.81 | Increased the risk of RA |
|  | Walker | 2009 | Canada | 1140/1248 | 3-1-2 | Y 0.51 | Increased the risk of RA |
|  | Takeuchi | 2006 | Japan | 100/104 | 3-1-2 | Y 0.23 | Not related |
|  | Barton | 2004 | UK | 151/152 | 3-1-2 | Y 0.31 | Not related |
|  | Lee | 2002 | Korea | 86/86 | 3-1-2 | N 0.01 | Increased the risk of RA |
|  | Gonzalez | 1999 | Spain | 138/305 | 3-0-2 | Y 0.41 | Not related |
| *CTLA-4* rs4553808 | Takeuchi | 2006 | Japan | 100/104 | 3-1-2 | Y 0.25 | Not related |
|  | Barton | 2004 | UK | 180/170 | 3-1-2 | Y 0.57 | Not related |
| *CTLA-4* rs16840252 | This study | 2018 | China | 573/792 | 3-1-2 | Y 0.5 | Not related |
| *CTLA-4* rs231779 | Walker | 2009 | Canada | 1248/1140 | 3-1-2 | Y 0.66 | Not related |
|  | Kim | 2010 | Japan | 298/406 | 3-0-2 | Y 0.24 | Not related |
| *CTLA-4* rs231777 | Kim | 2010 | Japan | 305/411 | 3-0-2 | Y 0.28 | Not related |
| *CTLA-4* rs11571297 | Walker | 2009 | Canada | 718/1140 | 3-1-2 | Y 0.66 | Increased the risk of RA |
| *CTLA-4* -1661A/G | Takeuchi | 2006 | Japan | 100/104 | 3-1-2 | Y 0.25 | Not related |
|  | Barton | 2004 | UK | 180/170 | 3-1-2 | Y 0.57 | Not related |
| *CTLA-4* rs733618 | Takeuchi | 2006 | Japan | 100/104 | 3-1-2 | Y 0.8 | Not related |
|  | Barton | 2004 | UK | 183/173 | 3-1-2 | Y 0.83 | Not related |
| *CTLA-4* rs231806 | Lei | 2005 | China | 326/250 | 2-1-2 | Y 0.07 | Not related |
| *CTLA-4* rs11571302 | Lei | 2005 | China | 326/250 | 2-1-2 | Y 0.23 | Not related |
| *CD80* 452G/A | Matsushita | 1999 | Japan | 101/168 | 3-1-2 | Y 0.67 | Not related |
| C*D86* rs17281995 | Liu | 2013 | China | 213/303 | 3-1-2 | Y 0.26 | Not related |
|  | This study | 2018 | China | 572/803 | 3-1-2 | Y 0.54 | Not related |
| *CD86* rs1129055 | Liu | 2013 | China | 213/303 | 3-1-2 | N 0.04 | Not related |
|  | Matsushita | 1999 | Japan | 101/168 | 2-1-2 | Y 0.73 | Not related |
| *CD86* rs2715267 | Lee | 2014 | Korea | 184/182 | 3-1-2 | Y 0.13 | Increased the risk of RA |
| *CD28* rs1980422 | Luterek | 2017 | Poland | 422/338 | 3-1-2 | Y 0.22 | Not related |
|  | Hegab | 2016 | Egyp | 385/394 | 3-1-2 | Y 0.1 | Increased the risk of RA |
| *CD28* rs3181097 | Kim | 2010 | Japan | 304/410 | 3-0-2 | Y 0.37 | Not related |
| *CD28* rs1879877 | Kim | 2010 | Japan | 298/406 | 3-0-2 | Y 0.52 | Not related |
| *CD28* rs2140148 | Kim | 2010 | Japan | 302/410 | 3-0-2 | Y 0.26 | Increased the risk of RA |
| *CD28* rs3116494 | Kim | 2010 | Japan | 307/411 | 3-0-2 | N 0.006 | Not related |

RA: rheumatoid arthritis.
