# Supplementary material for: The Association Between CTLA-4, CD80/86, and CD28 Gene Polymorphisms and Rheumatoid Arthritis: An Original Study and Meta-Analysis
Source: Front Med (Lausanne). 2021 Feb 2;8:598076. doi: 10.3389/fmed.2021.598076 (PMC7884472; doi:10.3389/fmed.2021.598076)
Supplement: Supplementary file 3 [file Table_3.DOCX]

**Supplementary table 3** Genotype distributions of *CTLA-4* gene polymorphisms in the included studies

| Gene & SNP | Author&Year | Ethnicity | Allele | | Case | | | Control | | | Genotype method |
| --- | --- | --- | --- | --- | --- | --- | --- | --- | --- | --- | --- |
|  |  |  | 1 | 2 | 11 | 12 | 22 | 11 | 12 | 22 |  |
| *CTLA-4* rs231775 | This study2018 | Asian | A | G | 303 | 206 | 63 | 387 | 342 | 68 | MassArray |
|  | Luterek2017 | Caucasian | A | G | 79 | 210 | 133 | 63 | 160 | 115 | TaqMan |
|  | Elshazli2015 | Caucasian | A | G | 14 | 55 | 43 | 6 | 45 | 71 | PCR-RFLP |
|  | Liu2013 | Asian | A | G | 77 | 111 | 25 | 130 | 125 | 48 | TaqMan |
|  | Alfadhli 2013 | Asian | A | G | 10 | 30 | 74 | 14 | 86 | 182 | PCR-RFLP |
|  | Tang2013 | Asian | A | G | 652 | 642 | 195 | 474 | 535 | 191 | TaqMan |
|  | Benhatchi2011. | Caucasian | A | G | 16 | 48 | 27 | 5 | 25 | 21 | PCR-RFLP |
|  | Plant (U)2010 | Caucasian | A | G | 146 | 451 | 407 | 410 | 1255 | 994 | MassArray |
|  | Plant (Gr)2010 | Caucasian | A | G | 26 | 133 | 113 | 33 | 107 | 147 | MassArray |
|  | Plant (Ge)2010 | Caucasian | A | G | 37 | 111 | 72 | 94 | 83 | 83 | MassArray |
|  | Plant (F)2010 | Caucasian | A | G | 96 | 315 | 273 | 15 | 75 | 72 | MassArray |
|  | Munoz2010 | Latin-American | A | G | 42 | 102 | 55 | 34 | 82 | 83 | PCR-RFLP |
|  | Walker2009 | Caucasian | A | G | 177 | 554 | 409 | 179 | 576 | 493 | MassArray |
|  | Tsukahara2008 | Asian | A | G | 636 | 668 | 186 | 181 | 194 | 73 | TaqMan |
|  | Suppiah2006 | Caucasian | A | G | 40 | 144 | 105 | 92 | 241 | 142 | PCR-RFLP |
|  | Takeuchi2006 | Asian | A | G | 49 | 39 | 12 | 44 | 49 | 11 | PCR-RFLP |
|  | Lei2005 | Asian | A | G | 148 | 138 | 40 | 86 | 125 | 39 | PCR |
|  | Barton2004 | Caucasian | A | G | 34 | 55 | 43 | 29 | 68 | 59 | TaqMan |
|  | Liu2004 | Asian | A | G | 14 | 42 | 9 | 21 | 50 | 10 | PCR-RFLP |
|  | Lee2003 | Asian | A | G | 103 | 67 | 16 | 85 | 100 | 18 | PCR-RFLP |
|  | Vaidya2002 | Caucasian | A | G | 20 | 65 | 38 | 45 | 158 | 146 | PCR-RFLP |
|  | Lee2002 | Asian | A | G | 41 | 35 | 10 | 49 | 29 | 8 | PCR-RFLP |
|  | Milicic2001 | Caucasian | A | G | 63 | 223 | 135 | 73 | 213 | 166 | PCR-RFLP |
|  | Hadj2001 | African | A | G | 23 | 27 | 10 | 68 | 62 | 20 | PCR-RFLP |
|  | Barton(1)2000 | Caucasian | A | G | 38 | 86 | 68 | 19 | 51 | 26 | PCR-RFLP |
|  | Barton(2)2000 | Caucasian | A | G | 14 | 57 | 65 | 12 | 70 | 62 | PCR-RFLP |
|  | Yanagawa2000 | Asian | A | G | 29 | 50 | 6 | 78 | 88 | 34 | PCR-RFLP |
|  | Gonzalez1999 | Caucasian | A | G | 10 | 63 | 65 | 30 | 103 | 172 | PCR |
|  | Matsushita1999 | Asian | A | G | 200 | 199 | 62 | 56 | 72 | 22 | PCR |
|  | Seidl1999 | Caucasian | A | G | 37 | 138 | 83 | 68 | 210 | 179 | PCR-RFLP |
| *CTLA-4* rs3087243 | Lutere2017 | Caucasian | C | T | 176 | 193 | 53 | 119 | 174 | 45 | PCR-RFLP |
|  | Torres2013 | Latin-American | C | T | 83 | 86 | 31 | 62 | 106 | 32 | PCR-RFLP |
|  | Gabalawy2011 | Caucasian | C | T | 126 | 161 | 45 | 198 | 226 | 66 | MassArray |
|  | Barton2009 | Caucasian | C | T | 1232 | 1760 | 677 | 892 | 1523 | 634 | MassArray |
|  | Walker2009 | Caucasian | C | T | 415 | 518 | 207 | 362 | 613 | 273 | MassArray |
|  | Costenbader2008 | Caucasian | C | T | 140 | 201 | 82 | 138 | 195 | 87 | TaqMan |
|  | Tsukahara2008 | Asian | C | T | 873 | 538 | 87 | 245 | 163 | 33 | TaqMan |
|  | Lei2005 | Asian | C | T | 156 | 137 | 33 | 87 | 131 | 32 | PCR |
|  | Plenge(1)2005 | Caucasian | C | T | 595 | 680 | 230 | 337 | 396 | 145 | MassArray |
|  | Plenge(2)2005 | Caucasian | C | T | 308 | 387 | 133 | 254 | 426 | 165 | MassArray |
|  | Orozco2004 | Caucasian | C | T | 117 | 198 | 118 | 101 | 199 | 98 | PCR-RFLP |
|  | Barton2004 | Caucasian | C | T | 242 | 336 | 141 | 239 | 370 | 146 | TaqMan |
| *CTLA-4* rs5742909 | Fattah2017 | Caucasian | G | C | 41 | 52 | 7 | 66 | 32 | 2 | PCR-RFLP |
|  | Torres2013 | Latin American | G | C | 182 | 16 | 2 | 180 | 20 | 0 | PCR-RFLP |
|  | Liu2013 | Asian | G | C | 102 | 97 | 14 | 214 | 77 | 13 | TaqMan |
|  | Walker2009 | Caucasian | G | C | 908 | 219 | 13 | 1055 | 183 | 10 | MassArray |
|  | Takeuchi2006 | Asian | G | C | 87 | 13 | 0 | 82 | 22 | 0 | PCR-RFLP |
|  | Barton2004 | Caucasian | G | C | 132 | 18 | 1 | 122 | 27 | 3 | TaqMan |
|  | Lee2002 | Asian | G | C | 65 | 19 | 2 | 68 | 14 | 4 | PCR-RFLP |
|  | Gonzalez1999 | Caucasian | G | C | 108 | 29 | 1 | 243 | 60 | 2 | PCR |
